# Pulmonary function, exhaled nitric oxide and symptoms in asthma patients with obesity: a cross-sectional study

**DOI:** 10.1186/s12931-017-0684-9

**Published:** 2017-12-07

**Authors:** Marise J. Kasteleyn, Tobias N. Bonten, Renée de Mutsert, Willemien Thijs, Pieter S. Hiemstra, Saskia le Cessie, Frits R. Rosendaal, Niels H. Chavannes, Christian Taube

**Affiliations:** 1 Department of Pulmonology, Leiden University Medical Center, V6-22, PO Box 9600, 2300 RC Leiden, the Netherlands; 2 Department of Public Health and Primary Care, Leiden University Medical Center, Leiden, the Netherlands; 3 Department of Clinical Epidemiology, Leiden University Medical Center, Leiden, the Netherlands; 4 Department of Pulmonary Medicine, Ruhrlandklinik, West German Lung Center, University Hospital Essen, University Duisburg-Essen, Essen, Germany

**Keywords:** Asthma, Obesity, Fe_NO_, Symptoms, Lung function

## Abstract

**Background:**

Obesity is a risk factor for the development of asthma. In patients with obesity the diagnosis of asthma is often based on symptoms, but without objective measurements. Nevertheless, obesity-associated asthma is recognized as a distinct asthma phenotype. Therefore, this study explores lung function and symptoms in asthma patients with and without obesity.

**Methods:**

The Netherlands Epidemiology of Obesity (NEO) study is a population-based cohort study with 6671 participants (aged 45–65 years) of whom 472 had asthma. Of this latter group, linear regression analysis was used to examine differences in lung function and symptoms between asthma patients with (*n* = 248) and without obesity (*n* = 224), and between asthma patients with and without increased Fe_NO_. Analyses were adjusted for confounders.

**Results:**

Asthma patients with obesity had lower predicted FEV_1_ and FVC values than patients without obesity [adjusted mean difference (MD) -3.3% predicted, 95% CI -6.5, −0.2; adjusted MD −5.0% predicted, 95% CI -7.8, −2.1]. The prevalence of symptoms was higher in patients with obesity. Asthma patients with obesity and with increased Fe_NO_ had lower FEV_1_ and FEV_1_/FVC values compared with those with low Fe_NO_ (adjusted MD −6.9% predicted, 95% CI -11.7, −2.0; −2.4%, 95% CI -4.6, −0.2).

**Conclusion:**

Asthma patients with obesity had lower FEV_1_ and FVC values than patients without obesity. This suggests that patients with obesity have restrictive lung function changes, rather than obstructive changes. Asthma patients with obesity and increased Fe_NO_ showed more obstructive changes. Fe_NO_ might help to identify patients with eosinophilic inflammation-driven asthma, whereas patients with low Fe_NO_ might have an obesity-associated asthma phenotype in which symptoms are partly caused by the obesity.

**Electronic supplementary material:**

The online version of this article (10.1186/s12931-017-0684-9) contains supplementary material, which is available to authorized users.

## Background

Asthma and obesity are common conditions with an increasing prevalence. It is estimated that, in 2014, worldwide more than 600 million adults had obesity [1]. In the general population, the prevalence of asthma ranges from 1 to 8% [2]. Epidemiological studies have shown that obesity itself has a significant impact on respiratory function [3] and some studies have suggested that obesity is also a risk factor to develop asthma [3–5]. Body mass index (BMI) has been shown to have little effect on spirometry results, while the expiratory reserve volume and functional residual capacity are reduced in people with obesity compared with people with normal weight [6]. Results regarding the relation between obesity and hyperresponsiveness are conflicting, with some indicating no relationship and others showing that BMI is associated with hyperresponsiveness [6].

Patients with and without obesity with a diagnosis of asthma differ in symptom severity, airway inflammation, age of asthma onset, sex, and treatment responsiveness. The Global Initiative for Asthma guidelines identify obesity-associated asthma as a distinct asthma phenotype [2, 7]. Nevertheless, asthma patients with obesity also represent a heterogeneous patient group regarding airway inflammation, symptoms and asthma control [8]. Obesity-associated asthma does not necessarily involve the classical type 2 T helper (Th2)-driven inflammatory process; also, it is suggested that the group of asthma patients with obesity is composed of patients with a Th2 inflammation-driven asthma and other patients who are mainly symptomatic as a result of their increased body weight without evidence of Th2-driven airway inflammation [9]. Indeed, especially in patients with morbid obesity and a diagnosis of asthma, airway inflammation was not detected in bronchial biopsies [10]. The detection of airway inflammation is important, as patients with increased eosinophilic inflammation should be treated differently than patients without evidence of eosinophilic inflammation. Fractional exhaled nitric oxide (Fe_NO_) has a diagnostic accuracy similar to blood eosinophils in identifying sputum eosinophilia in adult asthma patients [11]. Fe_NO_ measurement has emerged as a non-invasive, inexpensive and reliable test which is becoming available on a wider scale. Therefore, it may prove useful in the diagnosis and management of asthma in primary care, especially in patients with obesity. When evaluated at group level, Fe_NO_ is lower in asthma patients with obesity than in asthma patients without obesity, [12] suggesting that the group of patients with apparent airway inflammation is lower in patients with obesity. However, asthma patients with overweight or obesity have worse asthma control and respond less to corticosteroid therapy than normal weight individuals with asthma [13]. Other strategies, such as weight loss, improve pulmonary function, asthma control and quality of life in asthma patients with obesity, suggesting that the increased body weight and the resulting mechanical impairment of the lung might be an important factor [14, 15].

It can be questioned whether patients with obesity and with symptoms of dyspnea are adequately diagnosed as asthma. According to current international guidelines, the diagnosis asthma is based on medical history, physical examination, lung function tests and additional tests [2]. However, objective methods to confirm the diagnosis asthma are not always used; [16] in 30% of the adults with asthma, the diagnosis could not be confirmed by objective methods [17, 18]. Van Huisstede et al. suggest that, in obesity, respiratory symptoms could be incorrectly ascribed to either the obesity or asthma, resulting in a substantial proportion of misdiagnosis in this group [19]. Whereas the study of van Huisstede et al. suggests a difference in misdiagnosis of asthma between patients with and without obesity, Aaron et al. showed that overdiagnosis was not more common in patients with obesity than in those without obesity [20]. In 32% of the asthma patients with obesity, the diagnosis could not be confirmed by objective tests, compared with 29% of the patients without obesity [20].

To provide more evidence on the role of obesity in asthma, this study examines i) differences in pulmonary function, Fe_NO_ and symptoms between asthma patients with and without obesity, and ii) differences in lung function between asthma patients with and without increased Fe_NO_. The emerging knowledge may help optimize treatment in patients with obesity experiencing respiratory symptoms.

## Methods

### Study design and study population

The Netherlands Epidemiology of Obesity (NEO) study is a population-based prospective cohort study including 6671 men and women (aged 45–65 years), with an oversampling of persons with a BMI of ≥27 kg/m^2^. Details of the study design and data collection have been described previously [21]. Between September 2008 and September 2012 men and women aged 45–65 years, living in the greater area of Leiden (in the west of the Netherlands) and with a self-reported BMI of ≥27 kg/m^2^ were invited to participate. In addition, all inhabitants aged 45–65 years from one municipality (Leiderdorp) were also invited irrespective of their BMI.

Between 2012 and 2013 information on prescriptions and diagnoses was extracted from the electronic health records of the participants at their general practitioners. According to the guidelines of the Dutch College of General Practitioners, diagnoses are coded according to the International Classification of Primary Care (ICPC) [22]. In the patient records, the general practitioners link all contact moments to a care episode, which is coded with the ICPC. The information is listed under four categories: subjective, objective, evaluation and plan. Measurements are coded under the heading objective, ICPC codes under evaluation, and prescriptions under plan. Drug prescriptions in the electronic health records are coded according to the Anatomical Therapeutic Chemical (ATC) classification system [23].

The present study is a cross-sectional analysis of baseline measurements of all patients with an ICPC-coded diagnosis of asthma (R96) in their medical history (*n* = 513). Patients with missing values on the key outcomes were excluded (*n* = 41).

The Medical Ethical Committee of the Leiden University Medical Center (LUMC) approved the study protocol and all participants gave their written informed consent.

### Data collection in the NEO study

Participants were invited to a baseline visit at the NEO study center of the LUMC after an overnight fast. Prior to the NEO study baseline visit, participants completed a questionnaire about demographics, lifestyle, clinical information and quality of life. Participants reported their highest level of education in 10 categories according to the Dutch educational system. We defined low education as no education, primary education or lower vocational training. Self-identified ethnicity of participants was reported in eight categories that we grouped into Caucasian and other. Smoking status was categorized in to current, former or never.

The questionnaire included several questions about pulmonary symptoms. Participants indicated which symptoms (dyspnea, wheezing, coughing including mucus, coughing without mucus) they had experienced during the last month. They were also asked to indicate whether those symptoms worsened during physical activity, contact with animals, house dust mite or pollen, foggy weather, or at night and/or getting up in the morning. Physical activity during leisure time was reported using the Short Questionnaire to Assess Health-enhancing physical activity questionnaire and expressed in metabolic equivalents of task (MET) hours per week (MET h/week) [24]. Participants were asked to bring all medication they had used in the month preceding the study visit to the NEO study site, i.e. both prescribed medication and self-medication. All medications they were using at that time were checked and recorded by the study nurse. At the NEO study center, several measurements were performed including a physical examination and blood sampling. BMI was calculated by dividing the weight in kilograms by the height in meters squared. The World Health Organization defines obesity as a BMI ≥ 30 [1]. Duration of asthma was determined in two ways: i) as self-reported by the patient, and ii) calculated using the date of the first registration of the ICPC code asthma in the electronic health records. When using the registration date, the duration might be underestimated for patients with asthma since their youth, since electronic health records were introduced later. Therefore, in this study, we used the self-reported asthma duration; if this was missing, the date of the first registration of the ICPC code asthma was used.

### Pulmonary function tests

All participants performed pulmonary function tests, including measurement of Fe_NO_. These tests were performed at the Department of Pulmonology of the LUMC. Spirometry was performed according to the standards of the European Respiratory Society [25]. Testing was done in sitting position with nose clips in place. A spirometer (Jaeger Masterscreen PFT; Viasys Healthcare, Hoechberg, Germany) was used by trained technicians to measure forced expiratory volume in one second (FEV_1_) and forced vital capacity (FVC). The FEV_1_ and FVC values with the highest sum of FEV_1_ and corresponding FVC value of three technically satisfactory and reproducible curves were registered for analysis. Curves were considered reproducible if the FEV_1_ and FVC values were within 5%, and peak expiratory flow values within 10% of the highest values. When spirometric test results did not meet these criteria, but did include at least one technically acceptable curve, the values of this best performance were registered. FEV_1_% was defined as the FEV_1_ value expressed as a percentage of the predicted normal FEV_1_ and FVC% as the FVC value expressed as a percentage of the predicted normal FVC, both predictions based on sex, age and height. The Tiffeneau index (FEV_1_/FVC) was calculated by dividing the registered FEV_1_ value by the registered FVC value.

### Fe_NO_

Fe_NO_ was measured once using a portable analyzer, the NIOX MINO (Aerocrine AB, Solna, Sweden). We have previously shown that a single measurement suffices for the assessment of Fe_NO_ in persons with overweight or obesity [26]. In the present study, a cut-off of Fe_NO_ of <25 was used; this is a clinically relevant threshold for low Fe_NO_ according to the American Thoracic Society [27].

### Statistical analysis

Categorical variables were reported as numbers and percentages, normally distributed continuous variables as means with standard deviations (SD) and non-normally distributed continuous variables as medians with interquartile ranges (IQR), stratified by BMI (< 30 kg/m^2^ and ≥30 kg/m^2^). Differences in baseline characteristics between participants with obesity (BMI ≥ 30 kg/m^2^ and without obesity (BMI < 30 kg/m^2^) were tested, using linear regression analyses, separately for participants with and without an ICPC-coded asthma. For non-normally distributed variables and dichotomous variables, linear regression analyses with robust standard errors were used.

Interaction effects between the main independent variables (asthma and obesity, and obesity and Fe_NO_) were tested using linear regression analyses. For non-normally distributed variables and dichotomous variables linear regression analyses with robust standard errors were used. Interaction effects between obesity and asthma were tested in the total population with regard to lung function and Fe_NO_, and the symptoms were also tested. Furthermore, interaction effects between obesity and Fe_NO_ were tested in the asthma population. For the outcomes for which a significant interaction was found, the main effects were analyzed in the different subgroups.

Irrespective of the results of the interaction analyses, the asthma population was explored in more detail. In participants with asthma, differences in pulmonary function, Fe_NO_, symptoms and use of inhalation corticosteroids (ICS) between those with and without obesity were examined using linear regression analyses. In addition, differences in pulmonary function, symptoms and ICS use were examined between asthma patients with and without increased Fe_NO_ separately for those with and without obesity. In the case that the outcome was binary, robust standard errors were used to estimate risk difference. Adjustment for confounders was based on literature. Analyses were adjusted for age, sex, smoking, education levels, ethnicity, physical activity, alcohol use, and prescription of ICS. (Analyses for ICS use were not adjusted for prescription of ICS). These variables might act as confounders since they are associated with one of the main outcomes (obesity and/or Fe_NO_) and with one or more of the outcomes.

Analyses were performed with STATA Statistical Software (Statacorp, College Station, Texas, USA), version 12.0.

## Results

**Fig. 1:**
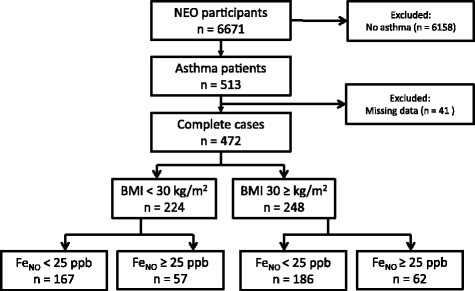
Flowchart. Fe_NO_: fractional exhaled nitric oxide; ppb: parts per billion

Of the 6671 participants, 637 were excluded due to missing values. Of the 6034 complete cases, 472 had an ICPC-coded diagnosis of asthma in their electronic health records (Fig. 1).

Baseline characteristics of the 472 included asthma patients (grouped according to BMI < and ≥30) are presented in Table 1. Of these 472 patients, 248 (52%) had a BMI ≥ 30 kg/m^2^. Most asthma patients were women (*n* = 287, 61%), the mean (SD) age was 55.1 (6.1) years and the mean (SD) BMI was 31.2 (5.3). The asthma patients with obesity had a significantly lower education, were less physically active, had a lower alcohol intake, and a higher BMI (Table 1). Baseline characteristics of the participants without asthma (grouped according to BMI < and ≥30) are presented in Additional file 1: Table S1.

**Table 1:** Baseline characteristics of asthma patients from the Netherlands Epidemiology of Obesity study (*n* = 472)

	BMI < 30 kg/m^2^	BMI ≥ 30 kg/m^2^	Difference (95% CI)^a^
	*n* = 224	n = 248	
Age in years, mean (SD)	55.4 (5.9)	54.9 (6.3)	−0.5 (−1.6, 0.6)
Sex, n (% men)	98 (44)	87 (35)	8.7 (−0.1, 17.5)
Height in meters, mean (SD)	1.7 (0.1)	1.7 (0.1)	−0.0 (−0.0, 0.0)
BMI in kg/m^2^, mean (SD)^b^	27.1 (2.4)	34.9 (4.4)	7.8 (7.2, 8.5)
Smoking			1.3 (−10.5, 13.2)
Never, *n* (%)	83 (37)	94 (38)	
Former, *n* (%)	117 (52)	122 (49)	
Current, *n* (%)	24 (11)	32 (13)	
Alcohol intake in g/d, mean (SD)^b^	15.6 (16.4)	11.4 (15.3)	−3.2 (−6.1, −0.3)
Ethnicity, *n* (% Caucasian)	208 (93)	235 (95)	1.9 (−2.5, 6.3)
Education, *n* (% high)	98 (44)	80 (32)	−11.5 (−20.3, −2.7)
Physical activity MET h/week, mean (SD)^b^	38.7 (35.5)	31.4 (30.8)	−7.3 (−13.4, −1.3)
Medication			
SABA, *n* (%)	34 (15)	49 (20)	4.6 (−2.3, 11.4)
LABA, *n* (%)	8 (4)	11 (4)	0.9 (−2.7, 4.4)
SAMA, *n* (%)	1 (1)	7 (3)	2.4 (1.2, 4.6)
LAMA, *n* (%)	12 (5)	12 (5)	0.5 (−4.5, 3.5)
ICS, *n* (%)	41 (18)	41 (17)	−1.8 (−8.7, 5.1)
Combination LABA + ICS, *n* (%)	71 (32)	84 (34)	2.2 (−6.3, 10.7)
Asthma duration in years, mean (SD)^b^	19.4 (18)	20.5 (18.1)	1.1 (−2.1, 4.4)

*BMI* body mass index, *g/d* gram/day, *MET* metabolic equivalent of tasks, *SABA* short-acting beta-agonists, *LABA* long-acting beta-agonists, *SAMA* short-acting muscarinic antagonists, *LAMA* long-acting β2-adrenergic antagonists, *ICS* inhalation corticosteroids

^a^Differences are presented as difference in means for continuous variables and difference in percentage for categorical variables

^b^Linear regression analyses with robust standard errors are used for dichotomous variables and non-normally distributed variables

In the total population, significant interaction effects between obesity and asthma were found for wheezing, worsening of symptoms during exercise, and worsening of symptoms during getting up in the morning. Differences in wheezing, worsening of symptoms during exercise and worsening of symptoms during getting up in the morning were more pronounced in participants with asthma compared with participants without asthma (Table 2). In the total population, no significant interaction effects between obesity and asthma were found for FEV_1_% predicted, FVC % predicted, FEV_1_/FVC, Fe_NO_, dyspnea, coughing with mucus, coughing without mucus and worsening of symptoms during dust/pollen/animal exposure, during foggy weather or at night. In the asthma population, no significant interactions were found between obesity and Fe_NO_.

**Table 2:** Main effects of significant interactions between asthma and obesity in the total population

	Participants without asthma				Participants with asthma			
	n = 5562				*N* = 472			
	BMI < 30 kg/m^2^ *n* = 3099	BMI ≥ 30 kg/m^2^ *n* = 2463	Unadjusted difference (95% CI)^a^	Adjusted difference (95% CI)^a^	BMI < 30 kg/m^2^ *n* = 224	BMI ≥ 30 kg/m^2^ *n* = 248	Unadjusted difference (95% CI)^a^	Adjusted difference (95% CI)
Wheezing, *n* (%)	120 (4)	192 (8)	3.9 (2.7, 5.1)	3.1 (1.9, 4.3)	39 (17)	88 (35)	18.1 (10.3, 25.9)	18.6 (10.8, 26.3)
Exercise, *n* (%)	259 (8)	324 (13)	4.8 (3.1, 6.5)	3.6 (1.9, 5.2)	77 (34)	122 (49)	14.8 (6.0, 2.4)	13.0 (4.2, 21.6)
Morning, *n* (%)	173 (6)	140 (6)	0.1 (−1.1, 1.3)	0.2 (−1.4, 1.1)	33 (15)	17 (7)	−7.9 (−13.5, −2.2)	−8.1 (−14.0, −2.2)

*BMI* body mass index, *CI* confidence interval

^a^Differences are presented as difference in percentage

Analyses are adjusted for age, sex, smoking, education levels, ethnicity, physical activity, alcohol use, and prescription of ICS

Differences in lung function and symptoms between asthma patients with and without obesity are reported in Table 3. Regarding lung function, asthma patients with obesity had lower predicted FEV_1_ and FVC values than patients without obesity. The prevalence of the symptoms dyspnea and wheezing was higher in patients with obesity than in those without obesity, and symptoms more often worsened during physical activity in the group with obesity than in those without obesity.

**Table 3:** Pulmonary function, Fe_NO_ and symptoms in asthma patients with and without obesity (*n* = 472)

	BMI < 30 kg/m^2^ *n* = 224	BMI ≥ 30 kg/m^2^ *n* = 248	Unadjusted difference (95% CI)^a^	Adjusted difference (95% CI)^a^
Lung function				
FEV_1_, % pred, mean (SD)	99.9 (18.1)	97.0 (17.1)	−2.9 (−6.1, 0.3)	−3.2 (−6.3, −0.03)
FVC, % pred, mean (SD)	111.8 (17.9)	107.4 (15.5)	−4.4 (−7.4, −1.4)	−4.9 (−7.8, −2.0)
FEV_1_/FVC, mean (SD)	74.0 (8.1)	74.9 (8.0)	0.9 (−0.5, 2.4)	0.5 (−0.9, 1.9)
Fe_NO_, ppb, mean (SD)	21.9 (16.9)	19.2 (13.9)	−2.7 (−5.5, 0.1)	−1.8 (−4.6, 1.0)
Symptoms				
Dyspnea, *n* (%)	78 (35)	114 (46)	11.1 (2.3, 20.0)	9.5 (0.8, 18.2)
Wheezing, *n* (%)	39 (17)	88 (36)	18.1 (10.3, 25.9)	18.5 (10.8, 26.2)
Coughing with mucus, *n* (%)	49 (22)	56 (23)	0.7 (−6.8, 8.3)	0.9 (−6.5, 8.4)
Coughing without mucus, *n* (%)	72 (32)	78 (32)	−0.7 (−9.1, 7.8)	−2.0 (−10.4, 6.3)
Symptoms worsen during:				
Physical activity, *n* (%)	77 (34)	121 (49)	14.8 (6.0, 23.7)	12.9 (4.2, 21.6)
Dust/pollen/animal exposure, *n* (%)	79 (35)	75 (30)	−5.0 (−13.5, 3.5)	−3.5 (−11.6, 4.5)
Foggy weather, *n* (%)	78 (35)	94 (38)	3.1 (−5.6, 11.8)	1.7 (−6.6, 9.9)
Night, *n* (%)	26 (12)	38 (15)	3.7 (−2.5, 9.9)	3.0 (−3.3, 9.3)
Getting up in the morning, *n* (%)	33 (15)	17 (7)	−7.9 (−13.5, −2.2)	−8.1 (−14.0, −2.2)
ICS prescription, *n* (%)	107 (48)	123 (50)	1.8 (−7.2, 10.9)	2.5 (−6.7, 11.7)

*BMI* body mass index, *CI* confidence interval, *FEV*_*1*_ forced expiratory volume in one second, *FVC* forced vital capacity, *Fe*_*NO*_ fractional exhaled nitric oxide, *ppb* parts per billion, *ICS* inhalation corticosteroids

^a^Differences are presented as difference in means for continuous variables and difference in percentage for categorical variables

Analyses are adjusted for age, sex, smoking, education levels, ethnicity, physical activity, alcohol use, and prescription of ICS

Of the asthma patients without obesity, 57 (25.4%) had Fe_NO_ levels of ≥25 ppb, whereas 167 (74.6%) had Fe_NO_ levels <25 ppb (Fig. 1). No differences in lung function, symptoms and ICS use were found between asthma patients without obesity, with and without increased Fe_NO_ (Table 4).

**Table 4:** Asthma patients without obesity: differences in pulmonary function between patients with and without increased Fe_NO_ (*n* = 224)

	Fe_NO_ < 25 ppb *n* = 167	Fe_NO_ ≥ 25 ppb *n* = 57	Unadjusted difference (95% CI)^a^	Adjusted difference (95% CI)^a^
Lung function				
FEV_1_, % pred, mean (SD)	101.5 (17.7)	95.2 (18.7)	−6.4 (−11.8, −0.9)	−4.4 (−9.7, 0.9)
FVC, % pred, mean (SD)	112.9 (17.9)	108.5 (18.5)	−4.4 (−9.7, 1.0)	−1.6 (−6.6, 3.4)
FEV_1_/FVC, mean (SD)	74.5 (8.0)	72.2 (8.4)	−2.3 (−4.8, 0.1)	−1.9 (−4.4, 0.6)
Symptoms				
Dyspnea, *n* (%)	60 (36)	18 (32)	−4.3 (−18.6, 9.9)	−1.1 (−15.4, 13.3)
Wheezing, *n* (%)	31 (19)	8 (14)	−4.5 (−15.4, 6.4)	−6.4 (−17.5, 4.8)
Coughing with mucus, *n* (%)	41 (26)	8 (14)	−10.5 (−21.8, 0.7)	−10.5 (−21.9, 1.0)
Coughing without mucus, *n* (%)	58 (35)	14 (26)	−10.2 (−23.6, 3.3)	−12.2 (−24.5, 0.1)
Symptoms worsen during:				
Physical activity, *n* (%)	62 (37)	15 (26)	−10.8 (−24.5, 2.9)	−11.1 (−24.2, 2.1)
Dust/pollen/animal exposure, *n* (%)	58 (35)	21 (37)	2.1 (−12.5, 16.7)	5.5 (−8.8, 19.8)
Foggy weather, *n* (%)	61 (37)	17 (30)	−6.7 (−20.8, 7.4)	−2.4 (−15.6, 10.7)
Night, *n* (%)	17 (10)	9 (16)	5.6 (−5.0, 16.2)	4.4 (−6.5, 15.3)
Getting up in the morning, *n* (%)	24 (14)	9 (16)	1.4 (−9.5, 12.4)	0.7 (−9.9, 11.4)
ICS prescription, *n* (%)	83 (50)	24 (42)	−7.6 (−22.7, 7.5)	−8.6 (−24.0, 6.7)

*Fe*_*NO*_ fractional exhaled nitric oxide, *CI* confidence interval, *FEV*_*1*_ forced expiratory volume in one second, *FVC* forced vital capacity, *ppb* parts per billion, *ICS* inhalation corticosteroids

^a^Differences are presented as difference in means for continuous variables and difference in percentage for categorical variables

Analyses are adjusted for age, sex, smoking, education levels, ethnicity, physical activity, alcohol use, and prescription of ICS

The majority of asthma patients with obesity had a Fe_NO_ value <25 ppb (Fig. 1). Differences between patients with and without increased Fe_NO_ are presented in Table 5. Patients with low Fe_NO_ levels had better FEV_1_ values and FEV_1_/FVC values than patients with increased Fe_NO_. In patients with higher Fe_NO_ levels, symptoms more often worsened during exposure to dust, pollen or animals compared with patients with lower Fe_NO_.

**Table 5:** Asthma patients with obesity: differences in pulmonary function between patients with and without increased Fe_NO_ (n = 248)

	Fe_NO_ < 25 ppb *n* = 186	Fe_NO_ ≥ 25 ppb *n* = 62	Unadjusted difference (95% CI)^a^	Adjusted difference (95% CI)^a^
Lung function				
FEV_1_, % pred, mean (SD)	99.0 (16.5)	91.2 (17.8)	−7.8 (−12.6, −2.9)	−6.9 (−11.9, −2.0)
FVC, % pred, mean (SD)	108.6 (15.2)	103.6 (16.0)	−5.0 (−9.4, −0.6)	−4.0 (−8.3, 0.4)
FEV_1_/FVC, mean (SD)	75.7 (7.6)	72.4 (8.7)	−3.3 (−5.6, −1.0)	−2.5 (−4.7, −0.2)
Symptoms				
Dyspnoea, *n* (%)	84 (45)	30 (48)	3.2 (−11.3, 17,7)	6.0 (−8.5, 20.5)
Wheezing, *n* (%)	66 (36)	22 (36)	−0.0 (−13.9, 13.9)	0.6 (−13.5, 14.7)
Coughing with mucus, *n* (%)	47 (25)	9 (15)	−10.8 (−21.6, 0.1)	−11.3 (−22.2, −0.4)
Coughing without mucus, *n* (%)	61 (33)	17 (27)	−5.4 (−18.5, 7.7)	−4.8 (−17.5, 8.0)
Symptoms worsen during:				
Physical activity, *n* (%)	95 (51)	27 (44)	−7.5 (−21.9, 6.9)	−4.5 (−18.9, 10.0)
Dust/pollen/animal exposure, *n* (%)	48 (26)	27 (44)	17.7 (3.8, 31.7)	16.2 (3.5, 28.8)
Foggy weather, *n* (%)	68 (37)	26 (42)	5.4 (−8.8, 19.6)	9.0 (−4.4, 22.3)
Night, *n* (%)	27 (15)	11 (18)	3.2 (−7.6, 14.1)	4.3 (−6.7, 15.2)
Getting up in the morning, *n* (%)	13 (7)	4 (7)	0.5 (−7.7, 6.7)	−1.2 (−8.7, 6.2)
ICS prescription, *n* (%)	93 (50)	30 (48)	−1.6 (−16.1, 12.9)	0.2 (−14.7, 15.1)

*Fe*_*NO*_ fractional exhaled nitric oxide, *CI* confidence interval, *FEV*_*1*_ forced expiratory volume in one second, *FVC* forced vital capacity, *ppb* parts per billion, *ICS* inhalation corticosteroids

^a^Differences are presented as difference in means for continuous variables and difference in percentage for categorical variables

Analyses are adjusted for age, sex, smoking, education levels, ethnicity, physical activity, alcohol use, and prescription of ICS

## Discussion

The aim of this study was to examine differences in pulmonary function, Fe_NO_ and symptoms between asthma patients with and without obesity, and to describe differences in lung function and symptoms between asthma patients with and without increased Fe_NO_. The results of this study suggest that: 1) differences between individuals with and without obesity in wheezing, worsening of symptoms during physical activity and worsening of symptoms during getting up in the morning are more pronounced in asthma patients compared with those without asthma, 2) patients with obesity and the diagnosis of asthma have decreased FEV_1_ and FVC values compared with asthma patients without obesity, 3) patients with obesity report more respiratory symptoms than asthma patients without obesity, and 4) asthma patients with obesity and with increased Fe_NO_ have lower FEV_1_ and FEV_1_/FVC values, and more symptoms due to environmental triggers, than patients with low FeNO levels.

In previous studies (and in our study), the use of spirometry to evaluate lung function in persons with morbid obesity revealed a proportional reduction in FVC and FEV_1_, suggesting the occurrence of restrictive lung function impairment [28]. The FEV_1_/FVC ratio is generally well preserved or elevated, even in individuals with morbid obesity, indicating that FEV_1_ and FVC are affected at the same rate [29]. Also in the present study, the FEV_1_/FVC ratio was not impaired in patients with obesity, suggesting no evidence for increased airway obstruction compared with patients without obesity.

In the present study, no difference was found in Fe_NO_ levels between asthma patients with and without obesity. This is in contrast with the studies of Komakula et al. [30] and Barros et al. [31] who reported a negative association between BMI and Fe_NO_ in asthma patients. Komakula et al. suggested that BMI may increase airway oxidative stress in asthmatics; this impact of oxidative stress on NO levels might explain the reported negative association [30]. Because we did not measure oxidative stress in the NEO study, we were unable to check its association with BMI in the present population.

In our study, patients with obesity reported more respiratory symptoms than asthma patients without obesity. Similarly, previous studies showed that individuals with overweight and obesity are more likely to have symptoms of wheezing and dyspnea than individuals with a normal BMI, even in the absence of demonstrable lung disease [32]. The origin of these symptoms is suggested to lie mainly with the overweight and not with airway obstruction [33, 34]. Because dyspnea can be attributed to either asthma or obesity, people with obesity are often misdiagnosed with asthma [19, 34]. Carpio et al. showed that a misdiagnosis of asthma in individuals with obesity could be attributed to an increased perception of dyspnea which, during exercise, is mainly associated with systemic inflammation and excessive ventilation for metabolic demands [35]. Their results confirm the suggestion that the main difference between subjects with obesity with misdiagnosed asthma and control subjects with obesity lies in a higher perception of dyspnea during the bronchial challenge and exercise, to such an extent that it is similar to that of asthmatic subjects.

Patients *without* obesity more frequently reported worsening of symptoms during getting up in the morning than patients with obesity. Because asthma symptoms fluctuate during the day and the presence of morning symptoms is common in asthma [36], one would expect such a worsening of symptoms in the morning in an asthma population. Therefore, it is remarkable that this was reported much more frequently by the asthma patients without obesity than by those with obesity, and that this difference was not found in the patients without asthma. This is an indication that asthma patients with obesity represent a specific group of asthma patients, in which symptoms are at least partly due to the overweight.

In patients both with and without obesity, the number of patients with increased Fe_NO_ levels was around 25%. Because increased Fe_NO_ values predict eosinophilic airway inflammation in both groups [11], we additionally stratified the analyses by high and low Fe_NO_ values. It can be speculated that increased Fe_NO_ reflects Th2-driven airway inflammation whereas patients with a low Fe_NO_ represent a phenotype in which symptoms are caused by their obesity rather than by airway inflammation and airway obstruction. However, it can also be argued that the difference between the two groups is due to the fact that patients in the low Fe_NO_ group were well controlled and adequately treated whereas patients in the high Fe_NO_ group were poorly controlled. Nevertheless, symptoms like dyspnea, wheezing and cough were present in similar numbers in asthma patients with increased or normal Fe_NO_ levels, suggesting that the disease control was similar in both groups. We observed that both obese and non-obese asthma patients with low Fe_NO_ levels more often reported cough with mucus than those with high FeNO. This is unexpected, also in view of a recent meta-analysis showing that high Fe_NO_ can be used to identify patients with cough-variant asthma in adult patients with chronic cough [37]. Our observations suggest that, within this asthma population, coughing with mucus is associated with a non-eosinophilic phenotype. Furthermore, coughing without mucus is relatively common in primary care, which might influence the results. In the group of asthma patients without obesity, there were no differences in lung function or symptoms between patients with high and low Fe_NO_. Interestingly, asthma patients with obesity and with increased Fe_NO_ had lower FEV_1_ values and FEV_1_/FVC compared with patients with lower Fe_NO_ levels, suggesting increased airway obstruction in this group. In addition, in patients with obesity and with increased Fe_NO_, symptoms more often worsened on exposure to pollen, dust or animals, suggesting the presence of an allergic component in this group. Therefore, the present study suggests that Fe_NO_ can be helpful to distinguish between Th2-driven asthma and obesity-associated dyspnea symptoms. Since the specificity of Fe_NO_ in the diagnosis of asthma is higher than the sensitivity, there is a higher potential for ruling in than for ruling out the diagnosis of asthma [38]. Nevertheless, in the absence of information about hyperreponsiveness, the question remains as to whether or not those individuals with the obesity-associated phenotype do in fact have asthma.

Obtaining a correct diagnosis of asthma in patients with obesity is important because, in the obesity-associated asthma group, other treatments (e.g. weight loss strategies) might be more efficient than pharmacological treatment. Indeed, weight loss in asthma patients with obesity can improve asthma control, asthma severity, lung function and quality of life, [14, 15] whereas it is refractory to standard asthma medications, including oral corticosteroids [39]. Nevertheless, in this population-based cohort, we observed that 50% of the asthma patients with obesity and with low Fe_NO_ levels were treated with ICS.

In addition to Fe_NO_, airway hyperresponsiveness assessed by methacholine challenge might also be used to differentiate between obesity-induced symptoms and symptoms due to asthma. Obesity, by itself, is not sufficient to alter airway responsiveness to methacholine in non-asthmatic subjects with normal airway responsiveness, although they do experience more dyspnea [40]. On the one hand, the use of Fe_NO_ could be preferred for the diagnosis of asthma patients with obesity, since this is less invasive than methacholine challenge testing. However, we cannot rule out that there is a group with hyperresponsiveness and without increased Fe_NO_ who might benefit from weight loss [15].

A strength of this study is that a large group of asthma patients (with an oversampling of individuals with overweight) were examined in detail, including information on pulmonary function, symptoms, FeNO levels and medication prescription. Also, for these patients ICPC-coded asthma was used, whereas most other studies rely on self-reported asthma.

A limitation of the study is that we have no information on several variables that can be used for the diagnosis of asthma, including reversibility testing, methacholine provocation testing, and blood or sputum eosinophils or immunoglobulin E. However, Fe_NO_ has a diagnostic accuracy comparable to blood eosinophils in identifying sputum eosinophilia in adult asthma patients, irrespective of the asthma phenotype such as severe, non-atopic, obese and smoking-related asthma [11]. Furthermore, because both pulmonary function and Fe_NO_ vary over time as a result of treatment, participants might have low Fe_NO_ levels during the study visit whereas at other moments their Fe_NO_ levels might be increased. Also, although both smoking and ICS can influence Fe_NO_ levels, all our analyses were adjusted for these potential confounders. Another limitation is the oversampling of people with a BMI ≥ 27 kg/m^2^_,_ which might hamper generalizability of the results to lean people. Nevertheless, in the present study, because the differences found between high BMI and low BMI are probably conservative, differences between the groups will likely be even more pronounced when considering normal weight versus obesity.

Future studies should focus on the effectiveness of using Fe_NO_ in the diagnosis of asthma in individuals with obesity, where high Fe_NO_ levels suggest Th2-driven asthma that should be treated with ICS, and low Fe_NO_ levels indicate obesity-associated symptoms for which intentional weight loss might be more effective. Also, the long-term effect of weight loss on asthma severity and control should be further investigated, since few studies have focused on long-term effects [41]. Alternative treatments should also be investigated, since early-onset obesity-associated asthma responds poorly to weight loss [42]. Finally, because obesity affects the respiratory system via changes in physiological mechanisms due to fat deposition in the chest wall, abdomen and upper airways (leading to symptoms of dyspnea), but also via systemic inflammation, more evidence on the association between mechanical and systemic processes and lung function is needed for a better understanding of the relationship between obesity and asthma.

## Conclusions

In conclusion, this study shows that these patients with obesity and the diagnosis of asthma have worse lung function and more symptoms compared with asthma patients without obesity. This suggests that patients with obesity have restrictive lung function changes, rather than obstructive changes. In addition, asthma patients with obesity and increased Fe_NO_ had lower FEV_1_ and FEV_1_/FVC values, and more symptoms due to environmental triggers, than patients with low FeNO levels. Fe_NO_ might help to differentiate between an asthma phenotype, where high Fe_NO_ identifies patients with eosinophilic inflammation-driven asthma and patients with low Fe_NO_ might have an obesity-associated asthma phenotype, in which symptoms are partly caused by the obesity. This knowledge might help to optimize treatment in patients with obesity who experience respiratory symptoms.

## Additional file

Additional file 1: Table S1. Baseline characteristics of participants without asthma from the Netherlands Epidemiology of Obesity study (*n* = 5562). (DOCX 12 kb)

## Abbreviations

ATC: Anatomical therapeutic chemical
Fe_NO_: Fractional exhaled nitric oxide
ICPC: International classification of primary care
ICS: Inhalation corticosteroids
LABA: Long-acting beta-agonists
LAMA: Long-acting β2-adrenergic antagonists
LUMC: Leiden University Medical Center
MET: Metabolic equivalents of task
NEO: Netherlands Epidemiology of Obesity
Ppb: Parts per billion
SABA: Short-acting beta-agonists
SAMA: Short-acting muscarinic antagonists
Th2: type 2 T helper
