# Supplementary material for: Pulmonary function, exhaled nitric oxide and symptoms in asthma patients with obesity: a cross-sectional study
Source: Respir Res. 2017 Dec 7;18:205. doi: 10.1186/s12931-017-0684-9 (PMC5719519; doi:10.1186/s12931-017-0684-9)
Supplement: Additional file 1: Table S1. — Baseline characteristics of participants without asthma from the Netherlands Epidemiology of Obesity study (n = 5562). (DOCX 12 kb) [file 12931_2017_684_MOESM1_ESM.docx]

**Additional file 1: Table S1. Baseline characteristics of participants without asthma from the Netherlands Epidemiology of Obesity study (n=5562)**

|  | **BMI < 30 kg/m^2^**  *n*=3099 | **BMI ≥ 30 kg/m^2^**  *n*=2463 | **Difference (95% CI)*** |
| --- | --- | --- | --- |
| Age in years, mean (SD) | 55.8 (6.0) | 55.8 (6.0) | -0.1 (-0.4, 0.2) |
| Sex, n (% men) | 1610 (52) | 1086 (44) | 7.9 (5.2, 10.6) |
| Height in meters, mean (SD) | 1.7 (1.0) | 1.7 (1.0) | -0.0 (-0.0, -0.0) |
| BMI in kg/m^2^, mean (SD)^$^ | 26.8 (2.6) | 33.9 (3.9) | 7.1 (6.9, 7.3) |
| Smoking |  |  | 3.0 (-0.7, 6.6) |
| Never, n (%) | 1120 (36.1) | 777 (31.6) |  |
| Former, n (%) | 1442 (46.5) | 1299 (52.7) |  |
| Current, n (%) | 537 (17.3) | 387 (15.7) |  |
| Alcohol intake in g/d, mean (SD)^$^ | 16.0 (17.0) | 14.2 (17.9) | -1.9 (-2.8, -0.9) |
| Ethnicity, n (% Caucasian) | 2971 (95.9) | 2340 (95.0) | -0.9 (-2.0, 0.2) |
| Education, n (% high) | 1392 (44.9) | 745 (30.3) | -14.7 (-17.2, -12.1) |
| Physical activity MET h/week, mean (SD)^$^ | 37.5 (33.3) | 33.1 (32.0) | -4.3 (-6.1, -2.6) |

BMI: body mass index; MET: metabolic equivalent of tasks; g/d: gram/day

* Differences are presented as difference in means for continuous variables and difference in percentage for categorical variables.

$ linear regression analyses with robust standard errors are used for dichotomous variables and non-normal variables.
